# Spatio-temporal analysis of land use land cover change and its impact on land surface temperature of Sialkot City, Pakistan

**DOI:** 10.1038/s41598-023-49608-x

**Published:** 2023-12-13

**Authors:** Kainat Javaid, Gul Zareen Ghafoor, Faiza Sharif, Memuna Ghafoor Shahid, Laila Shahzad, Naghmana Ghafoor, Muhammad Umar Hayyat, Muhammad Farhan

**Affiliations:** 1 Sustainable Development Study Centre, Government College University Lahore, Lahore, Pakistan; 2 Department of Botany, Government College University Lahore, Lahore, Pakistan; 3 Department of Economics, Lahore College for Women University, Lahore, Pakistan

**Keywords:** Climate change, Environmental impact

## Abstract

The dynamic interplay between urbanization and its impacts on climate is a subject of recent concern, particularly in rapidly urbanizing cities of Pakistan. This research investigated the spatio-temporal effects of urban growth in terms of Land Use Land Cover changes on the thermal environment (Land Surface Temperature) of the Sialkot city, Pakistan using satellite data spanning four distinct time periods (1989, 2000, 2009 and 2020) and predicted changes for year 2030 by employing Cellular Automata Markov Chain Model. Satellite imagery (Landsat 5, 7 and 8) was processed, and maximum likelihood supervised classification was done to generate LULC maps for each of the aforementioned years. In addition to LULC classification, thermal bands of satellite data (for summer and winter) were processed to compute Land Surface Temperature (LST) of the city. The prediction of LULC changes and LST was done for year 2030 using Cellular Automata Markov Chain Model. The accuracy of classified and prediction maps was checked using Kappa Index. The LULC analysis revealed 4.14% increase in the built-up area and 3.43% decrease in vegetation cover of the city during 1989 to 2020. Both land covers are expected to change in the future (year 2030) by + 1.31% (built-up) and − 1.1% (vegetation). Furthermore, a declining trend in the barren land and water bodies was also observed over time. These LULC changes were found affecting the LST of study area. The transformation of vegetation cover into built-up area resulted in an increase in LST over time. A notable rise of 4.5 °C (summer) and 5.7 °C (winter) in the mean LST of Sialkot was observed during 1989 to 2020 and further increases are anticipated in year 2030. This study calls for attention of the policy makers to reduce human impact on the local climate of the city. The study will also help city developers in analyzing the urban population growth trend, finding suitable location to built new infrastructure by governmental authorities and how the rising temperature can affect energy demand and agriculture production of the city in future.

## Introduction

The land use and land cover (LULC) changes and their impacts on the land surface temperature (LST) have emerged as a global environmental concern. While, the terms ‘land use’ and ‘land cover’ are sometimes used interchangeably; however, each has a distinct meaning. Land use refers to the utilization of land for various purposes such as recreation, education, housing and agriculture. On the other hand, land cover refers to the type of cover on the ground such as forest, bare rock or water^1^. LULC changes are related to urbanization, involving replacement of the natural environment (pervious surfaces) with the warm concrete based urban structures. These structures are known for their high heat absorbing capacities, thus affecting the land surface temperature of the cities^2^. The conversion of water surfaces and green spaces into barren land or built up areas lead to an increase in the LST. However, appropriate land use planning that minimizes the urban sprawl and promotes the green cover (by transforming barren areas) produces a cooling effect^3^.

Like near surface air temperature, the LST has been proposed as an essential climatic variable in the 2016 Global Climate Observing System Implementation Plan. The LST and air temperature are interrelated, hotter the surface, the higher the temperature of the surrounding air and vice versa^4^. LST data proves advantageous in situations where there is paucity in air temperature data due to a lack of weather stations. Additionally, satellite based LST data is more readily available for larger land areas aiding in urban planning^5,6^.

In twentieth century, the third world countries experienced rapid urbanization worldwide and are currently facing the consequences of high urban temperatures today^7^. The global urban population is projected to grow by 2.5 million in 2050, with a 90% increase expected in Asia^8^. The consequences of urbanization are not solely related to high land surface temperatures,growth in residential, transport and industrial sectors result in higher consumption of energy primarily from fossil fuels. This leads to high CO_2_ emissions causing global warming and other environmental degradations^7,9^. The expansion of concrete structures in the cities, combined with rising global air temperatures, poses increasing risks to urban populations and infrastructures in the face of climate change. Therefore, it is imperative to investigate the causes and effects of LULC changes on the local temperature of the urban areas in order to develop recommendations for climate change mitigation and city planning.

Based on remote sensing data, numerous studies have examined the relationship between LULC and LST. Tan et al.^10^ reported a 3.5 °C increase in the winter temperature of the Dongting Lake, China, due to the expansion of built-up and dryland area during 1995 to 2013. The Rajshahi district in Bangladesh experienced a 13 °C rise in LST resulting from LULC changes caused by urbanization during 1997 to 2017^11^. Traore et al.^12^ documented a 1 °C rise in the LST of Bangui city, Central Africa, as a result of a 130% expansion in built-up areas between 1986 and 2017. Ayanlade et al.^13^ estimated LST variations of 0.12 °C to 1 °C in four major cities in Nigeria due to vegetation loss, impervious cover increase and expansion of built-up space between 1984 and 2019. The LST of the Luis Potosí Basin, Mexico, increased by 11 °C during 2007 to 2020 due to LULC changes^14^. do Nascimento et al.^5^ reported higher surface temperatures (> 28 °C) in areas with high urban densities compared to land covers representing vegetation and water bodies (21 °C–25 °C) in the city of Sao Paulo, Brazil.

This study focuses on LULC transformations and its effect on the LST of an industrialized city in Pakistan i.e. Sialkot. Pakistan exhibits the highest pace of urbanization among South Asian nations with an annual urbanization rate of 2.4%^15^. Currently, 36% of Pakistan’s total population resides in urban areas, and it is projected to reach approximately 60% by 2050. Most of the Pakistani cities are highly dense with a huge urban population^16^. Consequently, urban areas in Pakistan are expanding in an unplanned manner to accommodate the growing urban population and meet their basic living needs. This uncontrolled expansion results in the proliferation of concrete structures such as residential and commercial buildings and roads, encroaching upon green spaces and other land uses leading to elevated urban temperatures. In province Punjab only, reduction of 1.77% in the green space and growth by 1.26% in the artificial surfaces with rise of 0.17 °C in LST has been documented during past two decades^17^.

The LST of Pakistan is also increasing due to LULC changes. However, there have been very few studies predicting the future effect of LULC on LST in Pakistani cities. The Gujranwala city experienced 8 °C rise in temperature due to urbanization during 2003 to 2018^18^. A 4 °C rise in the temperature of the Karachi city was reported due to growth of the built-up area during 1998 to 2018^19^. Mumtaz et al.^3^ documented a decrease in the LST in Peshawar city due to expansion of vegetation, while an increase in the LST was observed in Lahore city due to growth of the built-up area over a period of 20 years. The built-up area of the Khanewal city has grown from 1.75% in 1980 to 5.27% in 2020, resulting in a 0.5 °C rise in the LST during the last four decades^20^. Similarly, an increase of 3.5% in the built up area in the Multan city has contributed to an increase in LST (from 27.6 °C to 28.5 °C) during 1990–2020^21^.

Pakistan, in general, and Sialkot city in particular, lacks an appropriate city planning. Modeling based suitability analyses are generally not conducted during city expansion or development planning processes. The Sialkot city is undergoing rapid urbanization and is also becoming an industrial hub in the country attracting more people from surrounding under-privileged areas. The industries although adds to the economic development but also puts pressure on environment in terms of CO_2_ emissions thus contributing to the rise in temperature. The industrial developments and increased influx of migrants to seek opportunities is further leading to city expansion engulfing green spaces and agricultural land. The Sialkot city is economically significant in terms of its agriculture production with a total of 394,000 ha of cropped area contributing to the country’s food security which is declining due to uncontrolled expansions. Decrease in the cropped area and food production has been reported for Sialkot city over the course of time^22^. Therefore, analyzing the spatial patterns of this development (LULC), its role in changing LST and predicting the future trend in LULC and LST variation using machine learning techniques can help in reducing pressure on finite land resource of Sialkot city. Also most of the LULC assessments conducted yet are quantitative in nature, however novelty of this work lies in using machine learning techniques such as Cellular Automata and Markov Chain to better predict the spatial aspects of LULC and LST changes in future based on historical trends. However, to the best of our knowledge, no study has been conducted to analyze the effects of LULC changes on the climate of Sialkot city (economically significant for its industries and agricultural production), which could provide valuable insights for city planning. Therefore, the current study was designed with the objectives to (1) comprehensively analyze the spatio-temporal changes in LULC spanning the period from 1989 to 2020, (2) analyze the relationship between LULC transformations and their impact on the land surface temperature (LST) of Sialkot city during summer and winter and (3) employ a predictive modeling approach to predict likely changes in LULC and their corresponding impact on LST in the near future (year 2030) to provide insights into the evolving urban landscape and associated climatic implications.

## Methodology

### Study area

**Figure 1:**
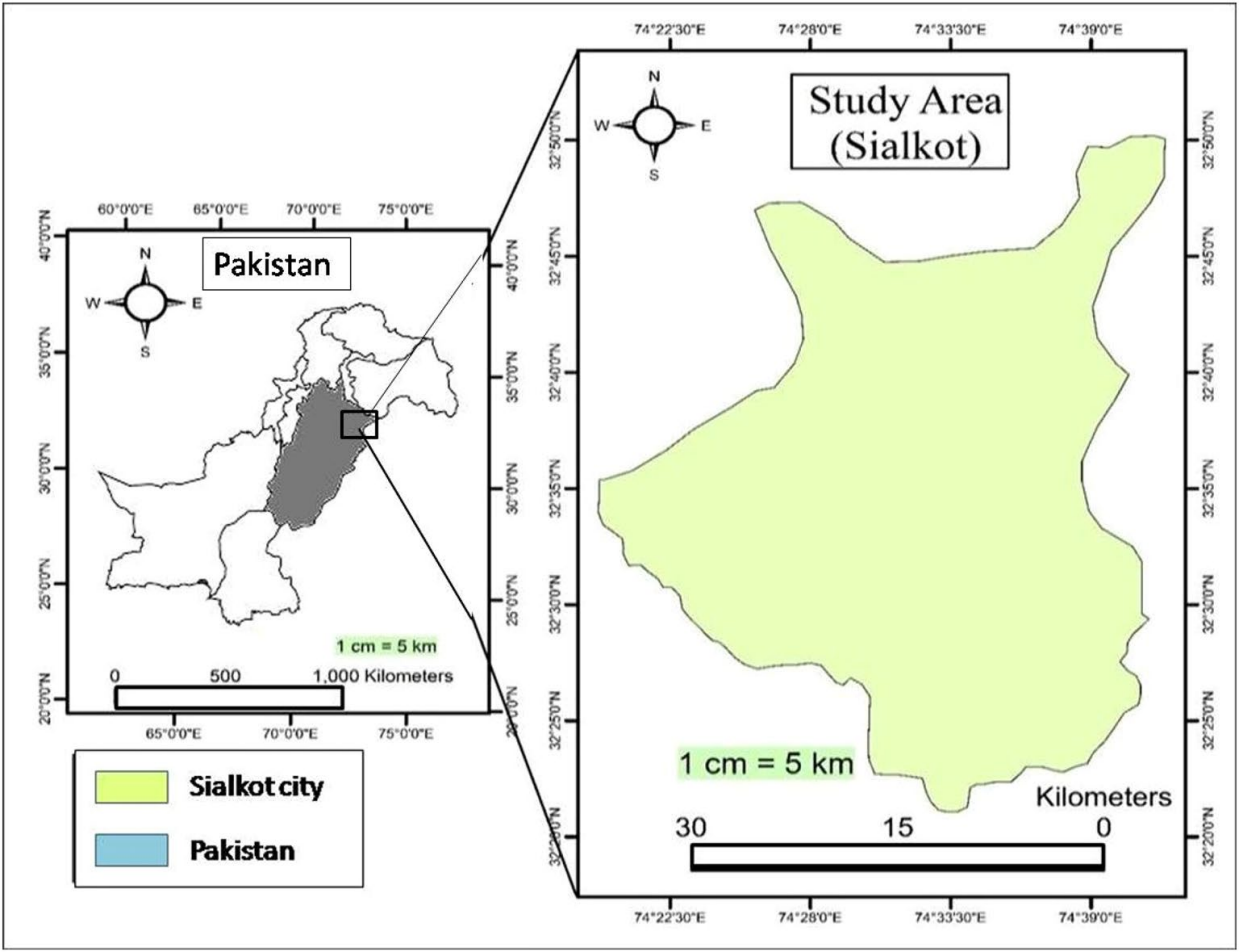
Map showing the location of the Sialkot city, Pakistan (Software: ArcMap v. 10.8).

This study was conducted in one of the industrial cities of Pakistan i.e., Sialkot city located at 32.4945° N and 74.5229° E, at an elevation of 256 m above sea level (m.a.s.l). The temperature of the city varies between 5 °C to 40 °C. The city is represented by built-up area surrounded by agricultural land and industries in the periphery. Common drains like Aik, Palkhu and Daik represent the water body surfaces in the city (Fig. 1). The location of the city is in the fertile agricultural basin in Punjab Province of Pakistan. The total Population of the Sialkot region was 655,852 as per census of 2017^22^. Industrial activities and migration from rural to the urban area are the reasons for the urban sprawl in the city. Most of the industries are in the outskirts of the city which attract migrants from nearby rural areas in search of better livelihood opportunities. The city has outgrown considerably since past due to rural–urban migration which is evident from expansion of housing societies in the periphery of the city engulfing fertile agriculture land.

### Data collection and pre-processing

Satellite imagery was processed to analyze the impacts of LULC changes on LST of the Sialkot city. Details of the satellite data downloaded and processed for analysis is given in Table 1. Satellite imagery was downloaded for the summer (May) and winter (February) months of 1989, 2000, 2009 and 2020 to analyze the effects of LULC on seasonal temperature variation. The difference in the date of image acquisition is related to the availability of satellite data and cloud cover^23^. While downloading satellite data for a specific date, meteorological and environmental conditions were considered to rule out any impact of rainy weather, climatic, anthropogenic, and biochemical factors on image quality. Atmospheric, radiometric and sun elevation corrections were done to normalize satellite data taken at different locations and times. The scan line errors in Landsat 7 data were removed using Landsat toolbox. For this purpose, raster data with scan lines was imported and Fix Landsat 7 Scanline Error tool was applied and scan lines were removed before further processing. The satellite data was then processed in the IDRISI SELVA (v. 17.0) to prepare LULC maps. ArcMap (v. 10.8) was used for the retrieval of LST from thermal bands. Prediction of LULC and LST for the year 2030 was also done in the IDRISI SELVA.

**Table 1:** Summary of the Landsat data collected for processing (at < 10% cloud cover).

Year	Sensor	Path/row	Summer data	Winter data	Bands for LULC	Band(s) for LST
1989	Landsat 5 TM	149/37	16 May 1989	09 Feb 1989	2 (Green), 3 (Red) and 4 (NIR)	6 (Thermal)
2000	Landsat 5 TM	149/37	14 May 2000	24 Feb 2000		
2009	Landsat 7 ETM	149/37	23 May 2009*	16 Feb 2009*		
2020	Landsat 8 OLI/TRIS	149/37	05 May 2020	15 Feb 2020	3 (Green), 4 (Red) and 5 (NIR)	10 (Thermal)

* The available 2010 imagery had high cloud cover, therefore, 2009 imagery was used.

### Land use land cover classification (LULC)

**Figure 2:**
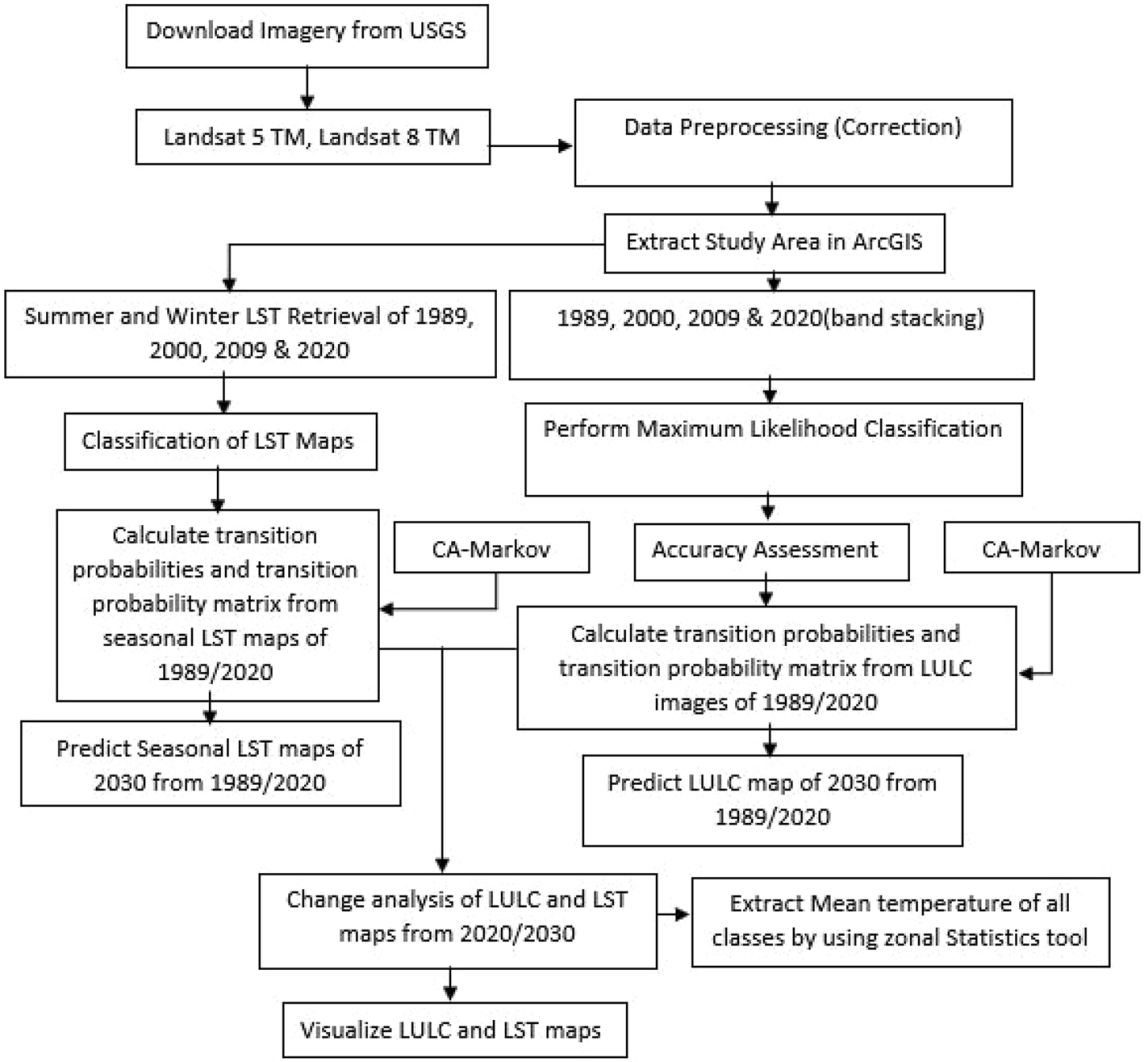
Methodological framework to develop LULC and LST maps.

Time series LULC maps of the Sialkot city were prepared for years 1989, 2000, 2009 and 2020 through supervised maximum likelihood classification. To classify LULC, bands of satellite imagery were stacked and a composite was made. For Landsat 5 TM, bands 2, 3, and 4 were used, while for Landsat 7 ETM and 8 OLI/TRIS, bands 3, 4, and 5 were used. A specific LULC class was assigned to each pixel of the composite raster. For this purpose, a representative number of training sample areas (polygons) of each LULC class was taken randomly and supervised maximum likelihood classification was used to classify the pixels representing each LULC class. To accurately select those training sample areas, a field survey was done initially and locations (latitude and longitudes) of the dominant land use land covers of the city were taken. During the field survey, dominant LULC classes identified included built-up area, barren land, water body and vegetation (Table 2). Approximately 70 GPS points were taken for the built-up area, 120 points for vegetation and 30 points each for the water-body and the barren land. In the IDRISI SELVA, the composite image was further processed using MAKESIG (Signature Extraction) tool for assigning a specific group number to all pixels having the same color (corresponding to each LULC class). The maximum number of training sample areas was taken for each class to ensure the accuracy of the classification process. The last step followed for maximum likelihood classification was to give the signature samples of all classes to a tool called MAXLIKE (Maximum Likelihood Classification). This tool selected all the pixels from the raster having the same values of a signature category or class. This tool produced the final classified map for further analysis (Fig. 2). The accuracy of the classified LULC map of year 2020 was assessed based on the ground truth data using the Kappa Index, user’s accuracy and overall accuracy methods following Hussain et al.^21^.

**Table 2:** Description of the feature selection for classification.

LULC class	Description/definition
Built-up	Area with settlements, residential buildings, commercial buildings and roads
Vegetation	Area representing any type of vegetation including cultivated and non-cultivated land/plantable land and urban green spaces
Water body	Drains/nullahs, canals and ponds
Barren land	Area representing bare soil and sand with no vegetation cover or settlements

### Retrieval of land surface temperature (LST)

Table 1 presents the details of thermal bands processed to retrieve LST for year 1989, 2000, 2009 and 2020. For the limitation of seasonal influences, the satellite images of the February and May months were used. Digital numbers (DN) from the Landsat data were converted into spectral radiance. Then radiance, brightness-temperature, and emissivity were measured using the metadata header file to extract the land surface temperature from the map of interest^3,23–25^.

The spectral radiance was calculated following Eq. 1.1where L ƛ is the spectral radiance (Wm^−2^sr^−1^ µm^−1^), L_maxƛ_ is the spectral radiance scaled to Q_CALmax_ (Wm^−2^sr^−1^ µm^−1^), L_minƛ_ is the spectral radiance that is scaled to Q_CALmin_ (Wm^−2^sr^−1^ µm^−1^), Q_CALmax_ is the maximum quantized calibrated pixel value corresponding to L_maxƛ_ in DN = 255. The minimum quantized calibrated pixel value (DN = 0) corresponding to L_minƛ_^3,26^.

The Top of atmospheric (TOA) spectral radiance was calculated following Eq. 2.

2 $${\text{TOA }}\left( {\text{L}} \right) \, = {\text{ ML}} \times {\text{QCAL}} + {\text{AL}}$$

where ML is multiplicative rescaling factor of specific-Band from the metadata. The QCAL corresponds to 10 bands and AL is the additive rescaling factor of specific-Band from the metadata^26^.

Equation 3 was used to calculate the brightness temperature (TB) as follows;

3 $$\mathrm{TB }= (\frac{{\text{K}}2}{(In\left(\frac{k1}{L}\right)+1)})-273.15$$

where K_1_ and K_2_ are the calibrations constant. For Landsat 5 TM sensor the K_1_ constant = 607.76 mW cm^−2^sr^−1^ µm^−1^ and K_2_ = 1260.56 K. T is the effective satellite temperature in Kelvin. And l is spectral radiance in Wm^−2^sr^−1^ µm^−1^^3^.

NDVI (Normalized difference vegetation index) was determined using Eq. 4.

4 $${\text{NDVI }} = \, \left( {{\text{NIR }}{-}{\text{ RED}}} \right)/\left( {{\text{NIR }} + {\text{ RED}}} \right)$$

where NIR is near infrared band and RED is red band. The NDVI values vary between − 1 to + 1 corresponding to barren land and high vegetation. The NDVI was required to calculate the PV (proportion of vegetation) which is highly related to the emissivity (ε). The PV was calculated using Eq. 5 and ε using Eq. 6^27^.

5 $${\text{PV }} = \, \left( {\left( {{\text{NDVI }}{-}{\text{ NDVI}}_{{{\text{min}}}} } \right)/\left( {{\text{NDVI}}_{{{\text{max}}}} {-}{\text{ NDVI}}_{{{\text{min}}}} } \right)} \right)^{{2}}$$

6 $$\varepsilon = {\text{mPv}} + {\text{n}}$$

where n = 0.004 and m = 0.986^27^. The LST was calculated using Eq. 7.

7 $${\text{LST }} = \, \left( {{\text{TB}}/\left( {{1 } + \, \left( {0.00{115 } \times {\text{ TB }}/{ 1}.{4388}} \right) \, \times {\text{ Ln}}\left( \varepsilon \right)} \right)} \right)$$

To validate LST, the results were compared with the meteorological data (1989 to 2020) taken from two weather stations operating in the Sialkot city. The LST was compared with the ambient air temperature (Ta) to check for the difference in the spatial and ground data. Later, to inspect the linkage between LULC changes and LST, four temperature classes were made for summer (16 °C–25 °C, 25 °C–35 °C, 35 °C–40 °C, 40 °C–54 °C) and winter months (7 °C–13 °C, 13 °C–16 °C, 16 °C–19 °C, 20 °C–28 °C). This method helped in detecting sprawl and squeezed each temperature (LST) class on the map for LULC changes to be analyzed for the study period. The classes for each LST map were made by selecting a manual range and then sprawl was assessed.

### Prediction of LST and LULC for year 2030

The Cellular Automata Markov Chain Model was used for prediction of LULC and LST (year 2030) under Business As Usual Scenario (BAU). The Markov model was implemented on 2009 and 2020 maps to make transition areas and the transition probability matrix between initial and final states and temporal changes in LST and LULC were predicted (Eq. 8). Spatial simulations were performed using Cellular Automata (CA) model, which decided the data between different states in time for itself and its neighboring cells/pixels through the contiguity filter and decided change in cells by rules (Eq. 9). The model applied a weight factor based on proximity of nuclear and neighboring cells. This weight factor was then combined with transition probabilities to predict the state of neighboring cells, ensuring that the LULC and LST predictions were not random decisions^3,28^. In the IDRISI Selva, the CA iterations were set to 11 corresponding to time interval, 5 × 5 contiguity filter and transition probability matrix (2009–2020) to predict changes in LST and LULC. The combined CA–Markov model simulated spatio-temporal changes in LST and LULC using transition probability matrix as input to cellular automata to predict the expected cover (year 2030) of LST and LULC (Fig. 2). The model’s validation involved predicting the LST and LULC map for the year 2020, using data from 2000 and 2009 as inputs. Its accuracy was subsequently assessed against a real (actual) map of 2020 using Kappa index. The value of Kappa coefficient was found to be > 0.80 with overall accuracy > 90%.

$${P}_{ij}=\left[\begin{array}{c}{P}_{11} \dots {P}_{1n}\\ \vdots \vdots \vdots \\ {P}_{n1} \dots {P}_{nn}\end{array}\right]$$

8 $$(0 \le Pij < 1\mathrm{ \,and\,}{\sum }_{j=1}^{n}Pij=1, i,j=\mathrm{1,2},....n)$$

9 $$S\left( {t + { 1}} \right) \, = P_{ij} \times \, S\left( t \right)$$

where Pij is the transition probability matrix and S is the state of land use at time t and t + 1.

### Zonal statistics

Zonal statistics tool was used to analyze the mean LST over each LULC class. LST changes due to increase or decrease in land cover area were measured. The tool extracted the area from LST map by comparing it with each LULC class. The mean, minimum and maximum values for land surface temperature over corresponding LULC class were calculated to analyze the impact of LULC on LST of the city during study period.

### Data analysis

Correlation between the mean LST and the air temperature (Ta) was performed for both seasons. Previous air temperature data of two weather stations was acquired from the metrological office of the Sialkot city. Mean values of the LST and Ta variables were used to develop the correlation graph in MS Excel (v. 2010).

## Results

### LULC changes

**Figure 3:**
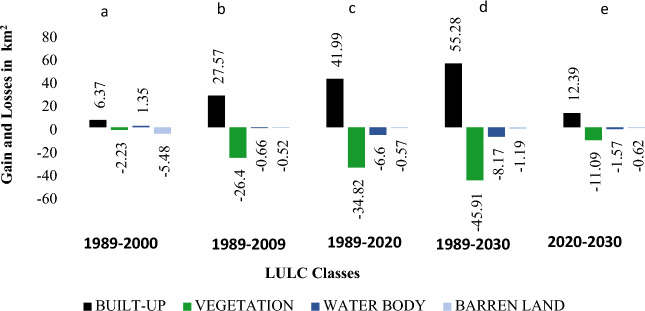
Gain and losses of LULC (**a**) 1989–2000. (**b**) 1989–2009. (**c**) 1989–2020. (**d**) 1989–2030. (**e**) 2020–2030.

**Figure 4:**
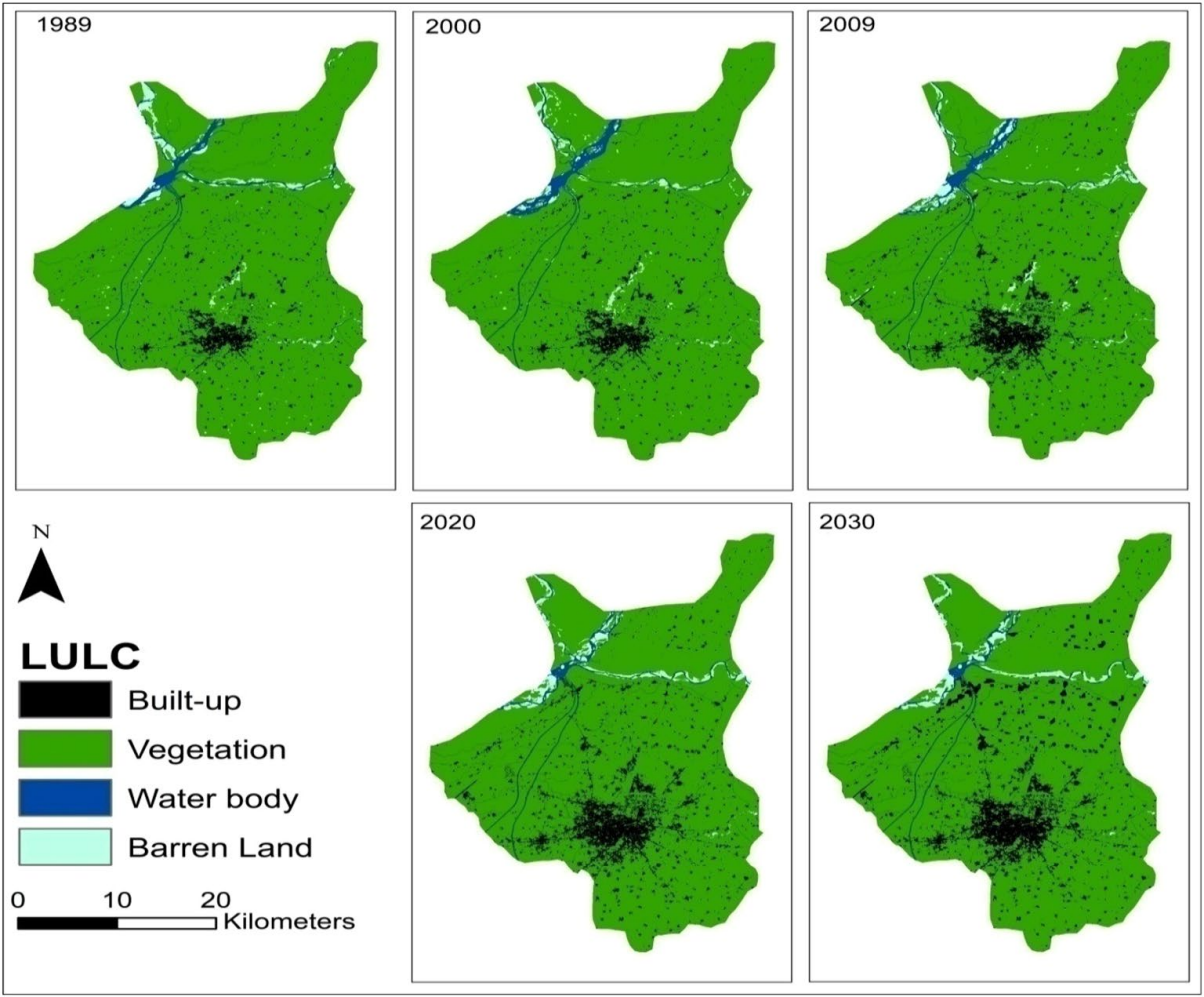
Spatio-temporal changes (1989–2030) in LULC of the Sialkot city, Pakistan (Software: ArcMap v. 10.8 & IDRISI SELVA v. 17.0).

Table 3 presents the changes in the cover area of each class during the baseline (1989, 2000, 2010, 2020 years) and predicted year (2030). It was observed that only built-up area was increased (+ 4.14%) in the baseline period while vegetation (− 3.53%), water body (− 0.7%) and barren land (− 0.05%) covers decreased during 1989 to 2020. The LULC predictions for 2030 also showed an increase in the built-up class by + 1.31% while decrease in vegetation (− 1.1%), water body (− 0.2%) and barren land (− 0.07%) than 2020. The gains and losses in LULC classes on decadal basis (in Km^2^) are presented in Fig. 3. Spatio-temporal changes in LULC during 1989 to 2030 are presented in Fig. 4. A noticeable increase in the built up area and decrease in the green cover of the city can be observed in the LULC maps. The prediction of LULC under BAU scenario also shows further growth (expected) in the built up area in the city engulfing other land uses in the year 2030. This conversion is expected both in the city center and in the outskirts representing expected urban sprawl in near future. There is a 15 min time lag in the equatorial crossing time of Landsat 5 and 8. However in the current study, there is little to no effect of this factor for temporal analysis (of LULC) with a gap of 20 years between two datasets (Landsat 5 and 8). As LULC changes are relatively slow and subtle hence 15 min time lag is unlikely to affect the analysis. The results of the accuracy assessment are available in supplementary material (Table SI 1 and 2; Fig. SI 1 and 2).

**Table 3:** Area Covered by LULC Classes with the Predicted year 2030.

Year	Area in ${\mathbf{K}\mathbf{m}}^{2}$ with (Percentage)			
	Built-up	Vegetation	Water body	Barren land
1989	35.95 (3.54)	927.27 (91.34)	30.54 (3.01)	21.45 (2.11)
2000	42.32 (4.17)	925.04 (91.12)	31.89 (3.14)	15.97 (1.57)
2009	63.52 (6.26)	900.87 (88.74)	29.88 (2.94)	20.93 (2.07)
2020	77.94 (7.68)	892.45 (87.91)	23.94 (2.36)	20.88 (2.06)
2030 (Predicted)	91.23 (8.99)	881.36 (86.81)	22.37 (2.20)	20.26 (1.99)

**Figure 5:**
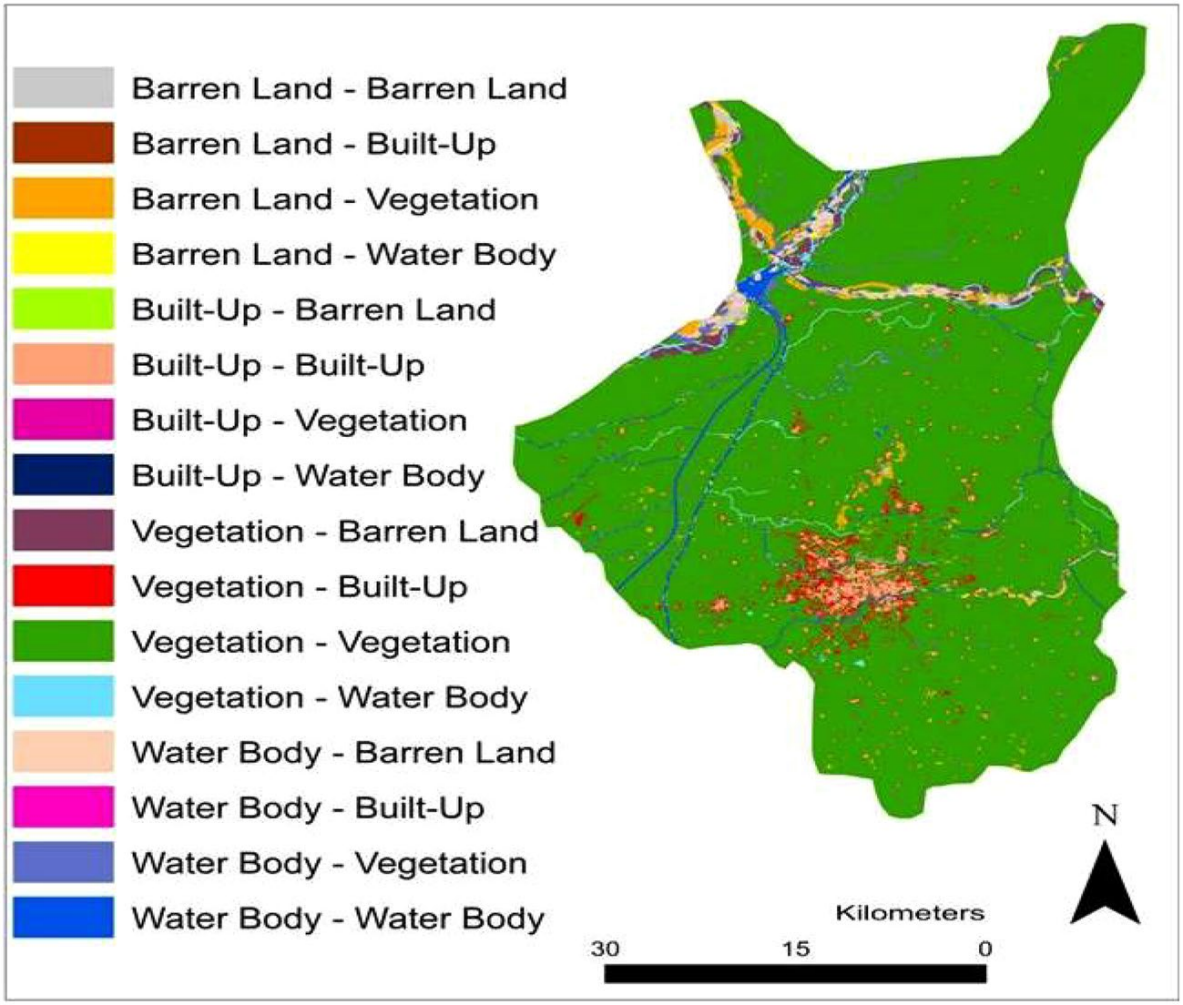
Transition of LULC between 1989 to 2020 (Software: ArcMap v. 10.8).

**Figure 6:**
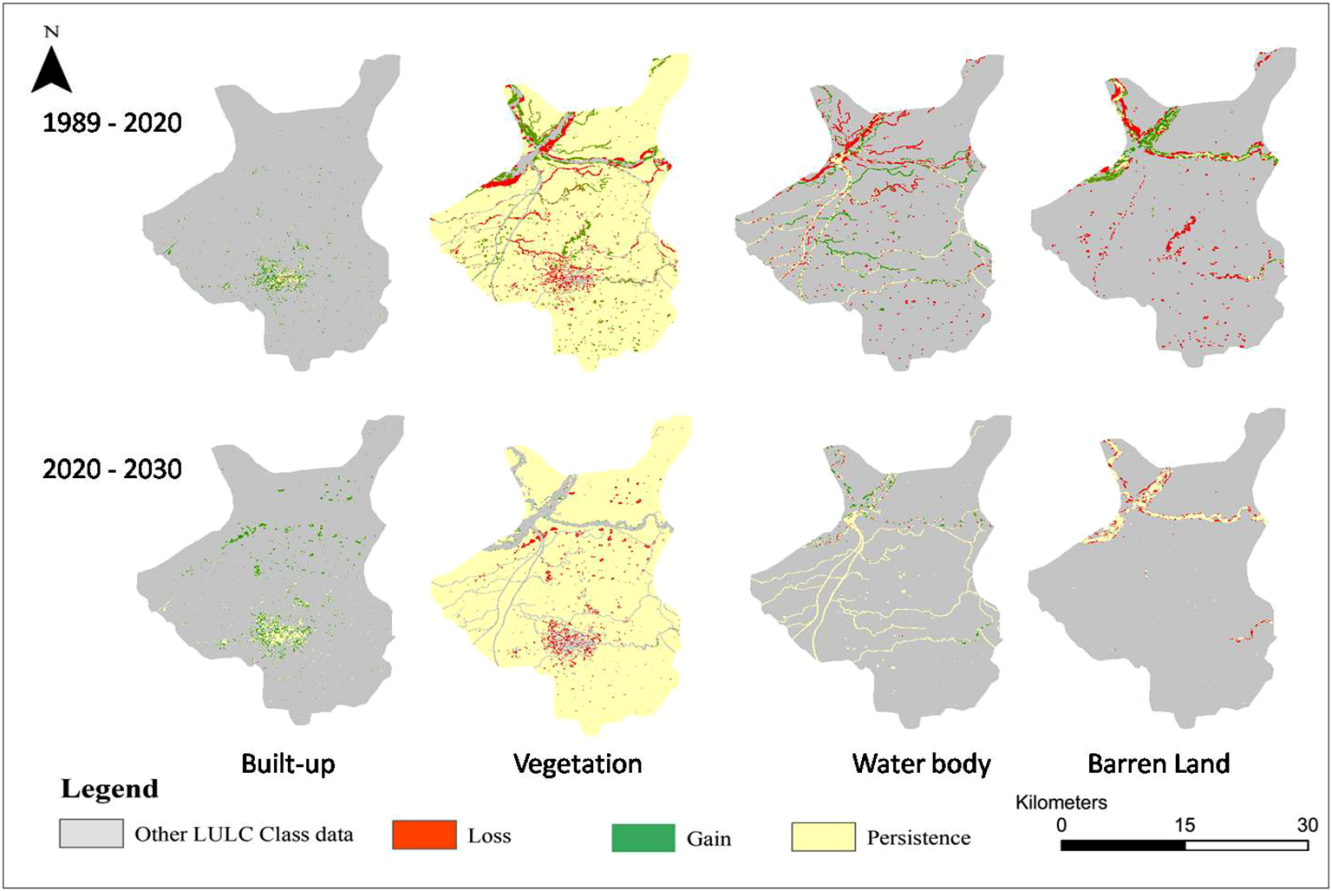
Location of the gain and loss in each of the LULC class during 1989–2020 and 2020–2030 (Software: ArcMap v. 10.8).

Figure 5 and Table 4 illustrates the inter-conversion or transition of LULC classes from 1989 to 2020. Approximately 41.05 Km^2^ of the vegetated area transformed into built up class, while 9 km^2^ of the vegetated areas converted into barren land. Additionally, 5 km^2^ of the area originally occupied by water bodies converted into barren land. Positive changes were also observed at certain locations, where vegetation replaced 11 km^2^ of water body and 13 km^2^ of the barren land, while 869 km^2^ area of the vegetation remained unchanged throughout the study period. Figure 6 depicts a detailed spatial representation (location) of gain and loss of each of the LULC class during historical (1989–2020) and prediction period (2020–2030).

**Table 4:** Inter-conversion of LULC classes and associated changes in area (1989–2020).

Change in LULC class	Area change (Km^2^)	Change in LULC class	Area change (Km^2^)
Built-up—built-up*	34.49982	Water body—vegetation	11.533631
Built-up—vegetation	0.671553	Water body—water body*	11.629763
Built-up—water body	0.016016	Water body—barren land	5.982018
Built-up—barren land	0.003223	Barren land—built-up	0.324418
Vegetation—built-up	41.045408	Barren land—vegetation	13.338637
Vegetation––vegetation*	869.051769	Barren land—water body	2.020843
Vegetation—water body	9.581048	Barren land—barren land*	5.462973
Vegetation—barren land	9.338002	Water body—built-up	0.581174

*Indicate no change in class over selected time period.

### LST changes

**Figure 7:**
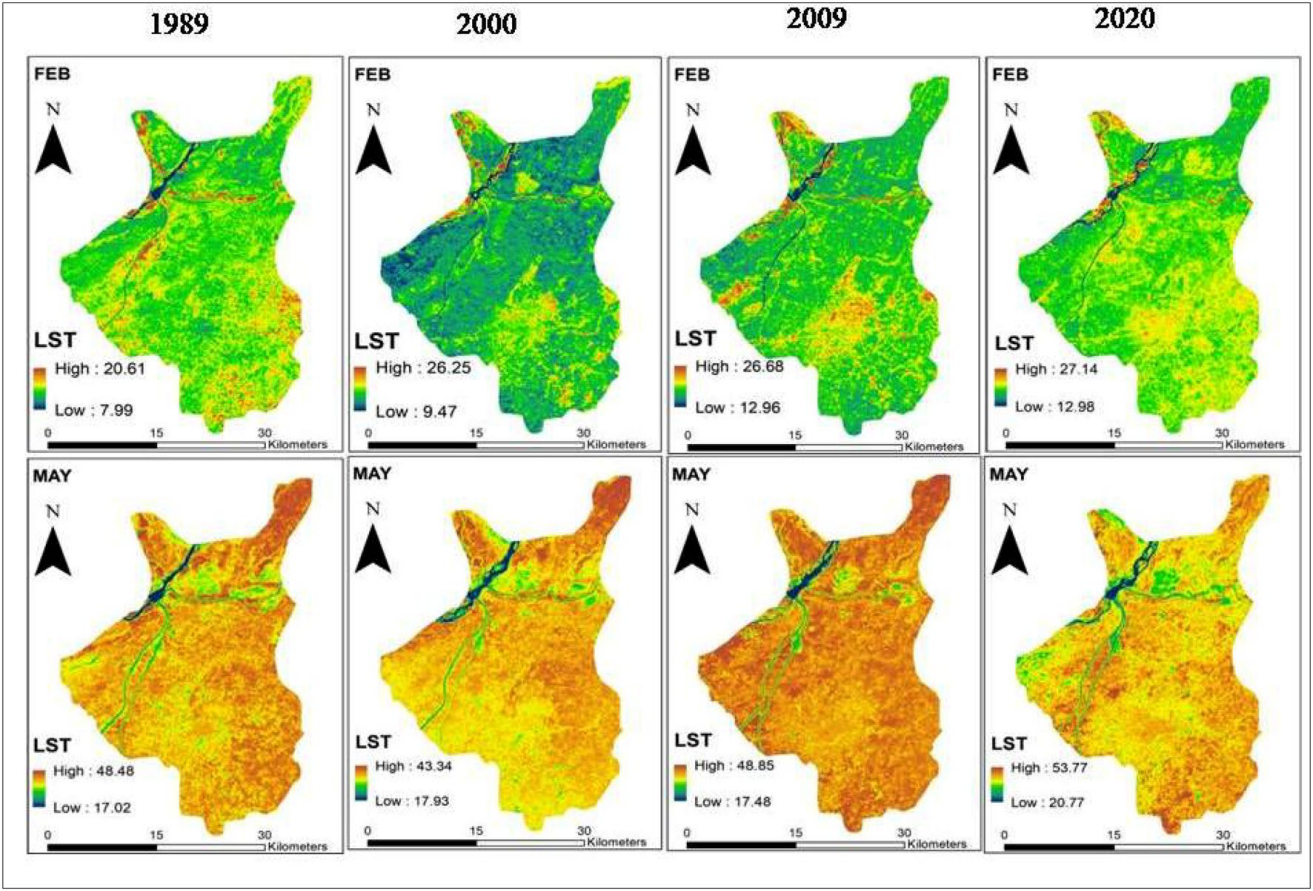
Changes in LST during winter (top) and summer (bottom) seasons in the Sialkot city (1989 to 2020) (Software: ArcMap v. 10.8).

Changes in the mean land surface temperature (LST) during 1989 to 2020 are presented in Table 5. The mean LST in summer increased from 32.75 °C to 37.27 °C (+ 4.52 °C) and in winter it increased from 14.30 °C to 20.06 °C (+ 5.76 °C) during 1989 to 2020. On a seasonal basis, comparatively higher temperature was perceived in the city center and lower on the outskirts during 1989 to 2020 (Fig. 7). Minimum to negligible chance of error might occur in this analysis due to difference in the crossing times of Landsat 5 and Landsat 8.

**Table 5:** Changes in LST (°C) during 1989 to 2020.

Year	Summer (°C)			Winter (°C)		
	Min Temp	Max Temp	Mean Temp	Min Temp	Max Temp	Mean Temp
1989	17.02	48.49	32.75	7.99	20.62	14.30
2000	18.43	50.45	34.44	9.47	26.25	17.86
2009	17.48	48.85	33.17	12.96	26.68	19.81
2020	20.78	53.77	37.27	12.98	27.14	20.06

**Figure 8:**
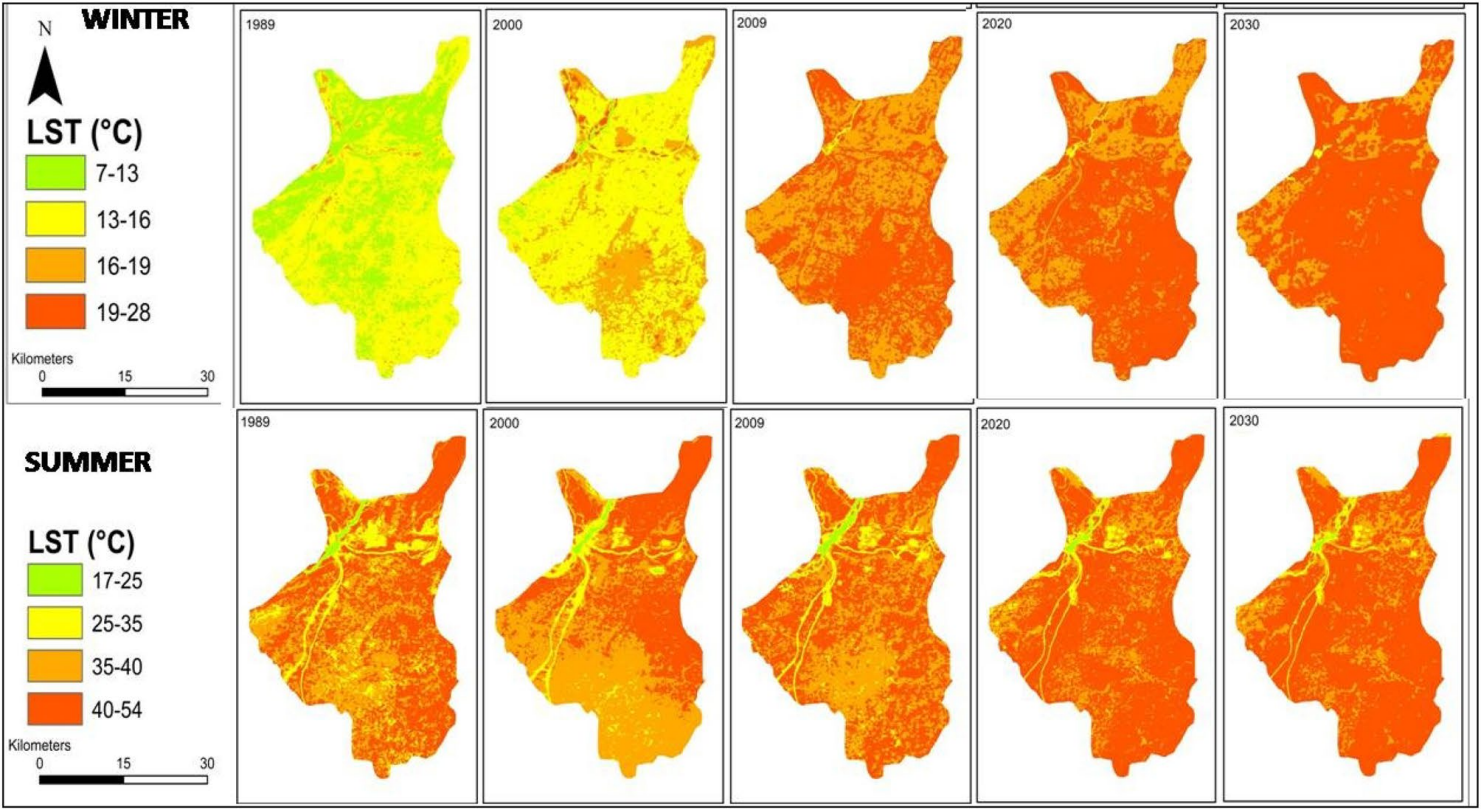
Changes in LST of Sialkot city during the baseline and predicted year (Software: ArcMap v. 10.8 & IDRISI SELVA v. 17.0).

Figure 8 and Table 6 present the area of the city covered by each LST class. An immense transformation in the summer temperature has been observed during the last 3 decades. Notably, there has been a decrease in the area covered by the first three summer LST classes (i.e. 16–25 °C, 25–35 °C, 35–40 °C), accompanied by an increase in the area covered by the highest class (i.e. 40–54 °C) during 2020 and the prediction year (2030), as compared to 1989. A similar trend of change in LST with respect to area has been observed for winter temperature, with decrease in the area covered by the 7–13 °C and 13–16 °C class and an increase in the area of higher temperature classes. The analysis suggests that in near future (2030), Sialkot city is likely to experience higher temperatures across most of its area.

**Table 6:** Baseline (1989 to 2020) and predicted LST changes (2030) with respect to the area of the city.

LST (°C)	Summer Area ${{\text{Km}}}^{2}$					LST (°C)	Winter Area ${{\text{Km}}}^{2}$				
	1989	2000	2009	2020	2030		1989	2000	2009	2020	2030
16–25	9.58	8.20	11.36	5.85	4.0	7–13	346.13	24.49	0.003	0.001	0.001
25–35	116.80	71.63	65.50	41.23	29.47	13–16	651.46	706.22	3.90	5.38	3.54
35–40	389.53	368.41	413.38	216.75	186.76	16–19	17.52	266.44	456.56	318.36	145.94
40–54	499.30	566.96	524.97	751.38	795.06	19–28	0.10	18.06	554.75	691.48	865.73

### Contribution of LULC to LST

**Figure 9:**
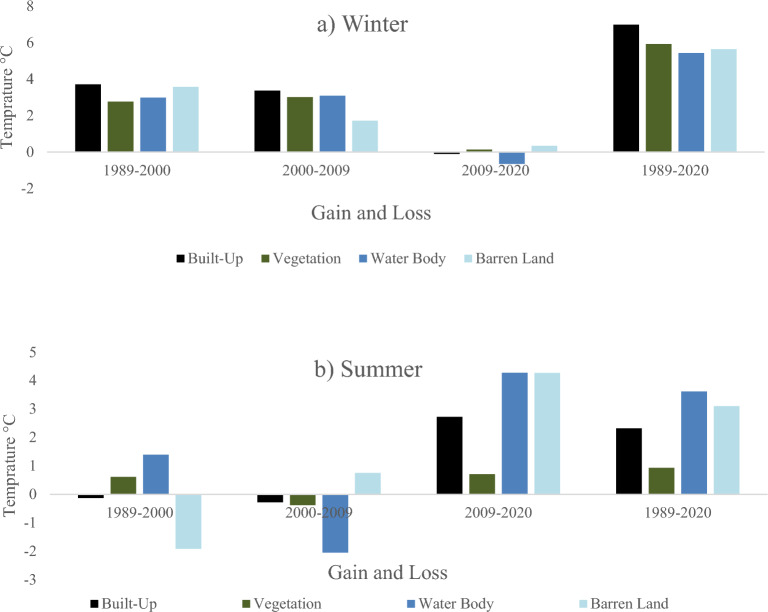
Temperature Gain and Losses of LULC Classes in both seasons. (**a**) Winter. (**b**) Summer.

Figure 9 presents the contribution of LULC transition to LST during summer and winter. Increase in the winter temperature was observed at all classes during study period (Fig. 9a). There was a gain of 6.9 °C in temperature in the built-up class when analysed for 1989–2020 transition indicating a decrease in winter season/days in the study area. Similarly, summer temperature has also increased in the study area. The built-up class was observed with a gain of 2.3 °C in temperature. The gain in temperature was also high for other LULC classes as well when analyzed for 1989–2020 transition (Fig. 9b).

### Zonal statistics

Table 7 presents zonal LST change observed on each LULC class. Notable during 1989–2020, the increase in LST was more significant in the winter season as compared to the summer season, reflecting the influence of LULC changes on local climate of the city. LST of barren land was high in both seasons because of the high reflectance characteristics of barren and sand surfaces. These results indicate probability of incidence of heat waves in Sialkot due to LULC changes. It was observed that builtup area contributed 31.2%, vegetation 2%, water body 26.1% and barren land 546% to the rise of summer temperature during 2009–2020. More pronounced changes in winter temperature occurred during 1989–2000 and 2000–2009 with high rate of contribution of LULC classes to LST rise.

**Table 7:** Seasonal Zonal Statistics of Mean LST (values in parenthesis indicate percentage rate of contribution of LULC transitions to LST during 1989–2000, 2000–2009 and 2009–2020).

Class	Summer Mean Temp (°C)				Class	Winter Mean Temp (°C)			
	1989	2000	2009	2020		1989	2000	2009	2020
Built-up	37.6	37.5 (− 0.3)	37.2 (− 0.3)	39.9 (31.2)	Built-up	12.40	16.12 (37.5)	19.49 (39.6)	19.39 (− 0.3)
Vegetation	38.3	38.9 (0.4)	38.5 (− 1.0)	39.2 (2.0)	Vegetation	12.87	15.64 (18.5)	18.66 (12.3)	18.80 (0.4)
Water body	27.9	29.3 (18.7)	27.3 (23.2)	31.5 (26.1)	Water body	11.85	14.84 (18.9)	17.94 (31.4)	17.29 (− 20.6)
Barren land	40.9	39.1 (20.1)	39.8 (45.3)	44.1 (546)	Barren land	14.90	18.48 (54.9)	20.21 (45.1)	20.55 (31.5)

### Correlation of LST with ambient air temperature (T_a_)

**Figure 10:**
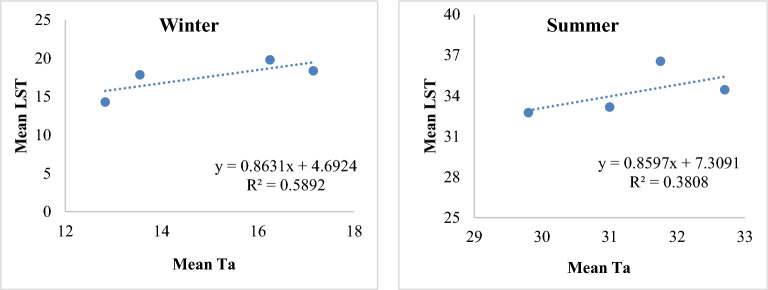
The correlation coefficient between winter and summer LST and T_a_ during 1989 to 2020.

To evaluate the Landsat derived LST trend, the values of land surface temperature (LST) and ambient air temperature (T_a_) were compared for both seasons. Table 8 shows the average increase of LST, T_a_, and the difference of LST from T_a_ during the baseline period (1989 to 2020) of both seasons. The mean difference between LST and T_a_ in winter and summer season was 2.64 °C and 2.91 °C respectively. The correlation coefficient comparison between LST and T_a_ for both seasons is shown in Fig. 10.

**Table 8:** Comparison of mean LST and mean T_a_.

Year	Temperature (°C)					
	Winter T_a_	Winter LST	Difference	Summer T_a_	Summer LST	Difference
2020	17.15	18.38	1.23	31.75	36.55	4.8
2009	16.25	19.81	3.565	31.0	33.16	2.16
2000	13.55	17.86	4.31	32.7	34.44	1.74
1989	12.83	14.31	1.48	29.8	32.75	2.95

## Discussion

The present study investigated the impact of urban sprawl on the land surface temperature (LST) of the Sialkot city, Pakistan. The findings of the study unveiled a notable increase in the LST of the city, particularly within built-up area, during the baseline and predicted time period. The study underscores that LULC changes have substantially increased the temperature of Sialkot city over the past 3 decades. Expansion of built-up areas and the reduction in vegetation cover have contributed to an increase of + 5.28 °C in the winter and + 6.52 °C in the summer temperature of the city during 1989 to 2020. This shows that development of concrete and impervious structures (built-up area) had absorbed more heat and increased the LST of the city during baseline period. Future prediction of the study showed that built-up area will further increased (+ 1.31%) and vegetation might decrease (− 1.1%) further affecting LST in 2030. Consistent to the findings of the current study, Kafy et al.^11^ also reported 7.24 °C rise in LST of Dhaka, Bangladesh due to 14% increase in built up area and 5% decrease in the vegetation cover during 2000 to 2020. Kafy et al.^11^ also documented a significant relationship between urbanization and LST of Rajshahi district, Bangladesh reporting 13 °C rise in temperature due to urban sprawl reducing vegetation cover during 1997–2017. In San Luis Potosi Basin, Mexico, the urban sprawl caused 11 °C rise in the LST during 2007 to 2020^14^. Differences in the LST estimates of current study and previous studies are related to the extent of land cover converted into built up area, study timeframe, local climate and month of satellite imagery processed for analysis.

In the current study, there was an increase of 2.32 °C in the LST within built-up area, 3.1 °C at bare land and 1 °C on vegetation during the baseline period. Traore et al.^12^ also reported an increase in LST of 1.38 °C for built up areas and 0.82 °C bare land during 1987–2017 in Bangui, Central Africa. Variations in LST among different LULC classes can be attributed to surface roughness and reflective and absorptive properties of solar radiations^29^.

The highest mean LST zone for Sialkot city was Barren land on each map because it includes the bare soil and sand. The surface of barren land is much hotter than any other class. Consequently, on every map the values of LST on barren land were high. In the summer, the maximum LST value of barren land is 8–10 °C higher than the built-up class. Zonal Statistics showed that the difference between the mean temperature of barren land and the built-up area was above 5 °C. Zonal statistics showed maximum LST values of the vegetation, water body, and barren land increased by 5.93 °C, 5.44 °C, and 5.65 °C respectively during study period. Ogunjobi et al.^30^ reported an increase of 2.9 °C and 4.9 °C temperature at built up and bare land in Sokoto Metropolis, Nigeria. The LST of built-up and bare soil area might be high as compared to surrounding area due to the thermal property of surface because bare surfaces absorb heat from sunlight and re-radiate it as thermal infrared radiation. Heat absorption also depends on the color of the soil and its moisture content as darker moist soils absorbs more radiant energy than the dry lighter colored soils.

The finding of this study showed that high-temperature classes covered most of the area of the city compared to 1989. Consistent to the findings of the current study, Sohail et al.^31^ also documented that most of the area of Lahore city in Pakistan was characterized by high temperature classes during 1980 to 2020. Similar trend was also reported by Tariq and Shu^23^ for the Faisalabad city in Pakistan during 1990 to 2018.

This urban sprawl is highly related to the climate of the Sialkot city. The findings of the spatial analysis for each year from 1989 to 2020 showed that the LST was slightly increased in both seasons due to the LULC changes in the last 3 decades. Similar trend has also been reported for other cities in Pakistan. Hassan et al.^32^ reported that Islamabad had faced the same changes in LST as observed during 1992–2012, the temperature trend was high with low precipitation and 1.52 °C LST increased due to LULC changes. Liaqut et al.^18^ reported that because of urban expansion and variations of rainfall patterns, the mean LST temperature of the Lahore city has increased by 3.1 °C during the past 20 years 1998–2018. Mumtaz et al.^3^ reported that the Gujranwala city has also faced an increase in LST (8 °C) due to urbanization during 2003 to 2018. This trend in LULC changes and LST can have significant impact on the local climate of an area. As urban cores are known for the urban heat island effect and further increase in the built-up area can increase heat in the city centers. This would warm surrounding air and might increase demand for energy to provide thermal comfort to the inhabitants in a subtropical region like Sialkot. Higher energy consumption is also related with increase in CO_2_ emissions which ultimately affects air temperature by making climate warm due to its heat trapping ability.

A strong correlation was also observed between Mean LST and Ta. The trend of the correlation was also positive for both seasons. Consistent to the findings of the current study, Mumtaz et al.^3^ also reported significant positive correlation between the LST of Lahore and Peshawar cities in Pakistan and their respective mean ambient temperature during 1980 to 2020.

The Sialkot city is an industrial and economic hub of Pakistan. The city faced a significant increase in industrial development during 1989 to 2019 that resulted in urban sprawl^33^. The main factor behind this urban sprawl in Sialkot was the unplanned development from provincial and local governments. There are around 56 new private housing schemes which are under development and most of those are illegal. These housing schemes were existing along the main roads of the city. That can be considered as a significant reason behind the land-use change in Sialkot city^34^.

LULC changes have occurred in the Sialkot city. It is the 12th most populated city in Pakistan with a total population of 3,894,000 in 2017 growing at a rate of 1.95%^22,35^. The consequences of this population surge, combined with pull factors of urbanization in the city has led to growth of factories, increased generation of solid waste and associated environmental concerns^36^. There were total 215 factories in the Sialkot city in year 2011, while the number escalated to 1082 in 2018. The number of registered vehicles increased from 268,186 in year 2008 to 696,825 in 2020^33,37^. With this growth in industries and housing societies, Sialkot has witnessed decline in its cropped area from 424,000 ha in year 2002 to 340,000 ha in 2020. Area sown for wheat production has also decreased from 193,000 ha in 2000 to 171,000 ha in 2021^38,39^.

A significant change has been observed from 1989 to 2020 in the pre-monsoon, monsoon, and post-monsoon pattern of Sialkot. The city has faced reduction in the monsoon precipitation during 1989 to 2020. The trend of change in the mean temperature and precipitation has also been observed for the whole country. Overall, Pakistan is experiencing reduction in monsoon rainfall^40–42^. Pakistan has already suffered 0.6 °C rise in the mean temperature however, further 1.1 °C to 6.4 °C rise is expected by the end of this century^43^. This trend in climatic changes for Pakistan coupled with LULC changes might result in significant rise in LST across the country in the future. The CA–Markov model used in this study could not perform predictions of the minimum, maximum and mean LST. But, LST classification was performed to analyze the spread of heat waves in relation to the covered area of the city. Further studies, employing more advanced models, can be carried to predict the likely impacts of LULC changes on LST and rainfall pattern of other cities or at country level. Such studies can provide valuable insights to the policy makers in making informed decisions on urban expansions in order to mitigate the likely impacts of climate change.

## Conclusion

This research provided a spatio-temporal analysis of land use land cover (LULC) changes and their subsequent impact on the LST of the Sialkot city from 1989 to 2020. The findings of this study reveal notable LULC changes in Sialkot during the specified period (1989–2020) influencing the local temperature of the city. The findings indicated 3.43% decrease in the vegetated land and 4.14% growth in the built-up area and barren land overtime and further expansion in the city’s buildup area is also expected in the near future (2030). The burgeoning urbanization and expansion of barren land has increased the land surface temperature of the city. The seasonal LST of Sialkot city has substantially increased in summer (4.5 °C) and winter (5.7 °C) in the last 3 decades. Projections indicate that anticipated growth in the built up and barren land in the future (year 2030) might lead to further increase in the LST of the city. Considering the imminent threat of urban sprawl on Sialkot’s local climate, this study can serve as a valuable resource for relevant authorities to make informed decisions. As a large area of the Sialkot is expected to face the pressure of urban sprawl in the future, so this study will help relevant authorities in identifying areas where policy interventions could be taken to reduce the impacts of urbanization on local climate variability such as through the creation of urban green spaces. The study will also help government in identifying areas with inappropriate land use planning. The city administration must make policies to curb urban sprawl, create green spaces in the city and make developments more climate-friendly.

### Supplementary Information

Supplementary Information.

## Data Availability

The data generated and analyzed in this study is included in this article and in supplementary material.
