# Supplementary material for: Spatio-temporal analysis of land use land cover change and its impact on land surface temperature of Sialkot City, Pakistan
Source: Sci Rep. 2023 Dec 13;13:22166. doi: 10.1038/s41598-023-49608-x (PMC10719351; doi:10.1038/s41598-023-49608-x)
Supplement: Supplementary file 1 — Supplementary Information. [file 41598_2023_49608_MOESM1_ESM.docx]

# Spatio-temporal analysis of land use land cover change and its impact on land surface temperature of Sialkot City, Pakistan

Kainat Javaid^a^, Gul Zareen Ghafoor^a*^, Faiza Sharif^a^, Memuna Ghafoor Shahid^b^, Laila Shahzad^a^, Naghmana Ghafoor^c^, Muhammad Umar Hayyat^a^, Muhammad Farhan^a^

^a^Sustainable Development Study Centre, Government College University Lahore, Pakistan

^b^Department of Botany, Government College University Lahore, Pakistan

^c^Department of Economics, Lahore College for Women University Lahore, Pakistan

(*Corresponding Author: [zareen.sdsc@gcu.edu.pk](mailto:zareen.sdsc@gcu.edu.pk), +92-343-4624022. <https://orcid.org/0000-0003-4784-3366>

**Supplementary material**

**Table SI 1.** Error matrix and Overall Accuracy of LULC Map of 2020

| Class | Built-Up | Vegetation | Water Body | Barren Land | User's Accuracy % |
| --- | --- | --- | --- | --- | --- |
| Built-Up | 38 | 3 | 0 | 0 | 92.68 |
| Vegetation | 2 | 36 | 0 | 0 | 94.74 |
| Water Body | 0 | 2 | 18 | 1 | 85.71 |
| Barren Land | 0 | 1 | 1 | 7 | 77.78 |
| Kappa Coefficient (T) | | | 0.87 | | |
| Overall Accuracy | | | 91.54 | | |

**Table SI 2.** Error matrix and Overall Accuracy of LULC Map of 2030

| Class | Built-Up | Vegetation | | Water Body | Barren Land | User's Accuracy % |
| --- | --- | --- | --- | --- | --- | --- |
| Built-Up | 37 | 3 | 0 | | 1 | 90.24 |
| Vegetation | 2 | 34 | 2 | | 0 | 89.47 |
| Water Body | 2 | 3 | 15 | | 1 | 71.42 |
| Barren Land | 0 | 2 | 1 | | 6 | 66.66 |
| Kappa Coefficient (T) | | | 0.75 | | | |
| Overall Accuracy | | | 80 | | | |


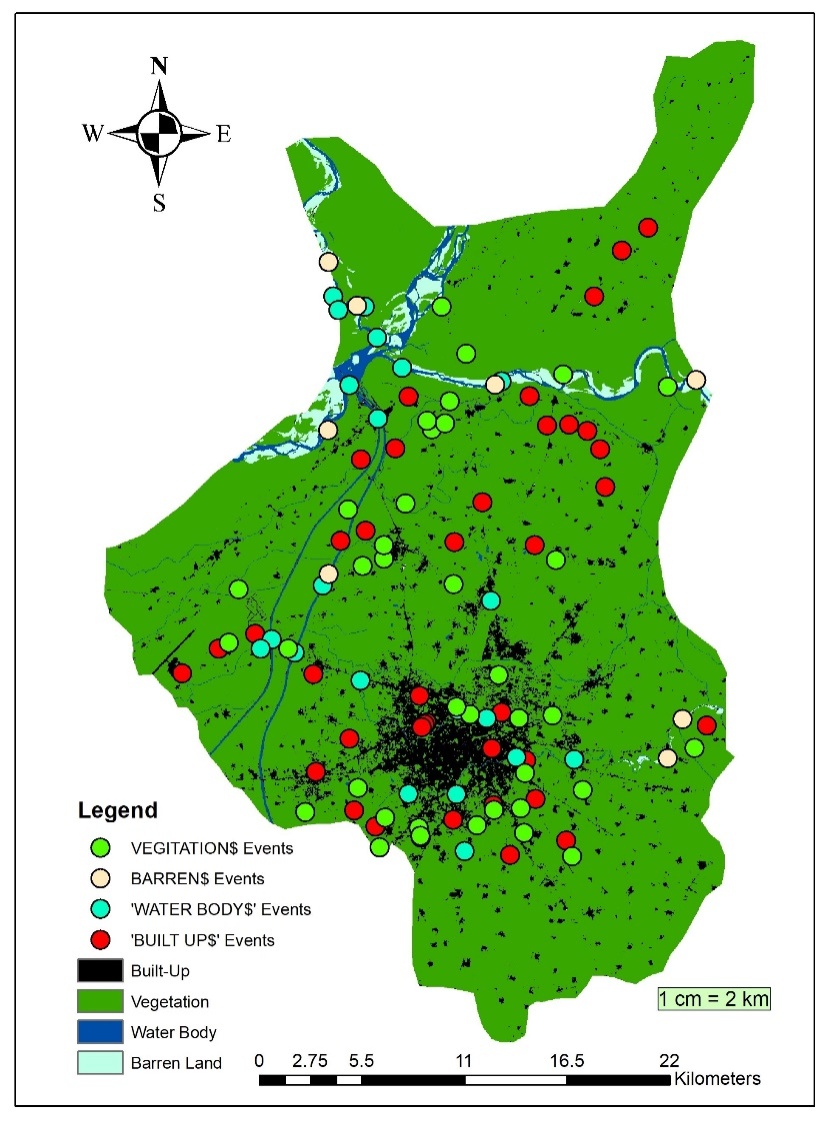


**Fig. SI 1.** Ground truth data for accuracy assessment of 2020 map (Software: ArcMap v. 10.8 & IDRISI SELVA v. 17.0)


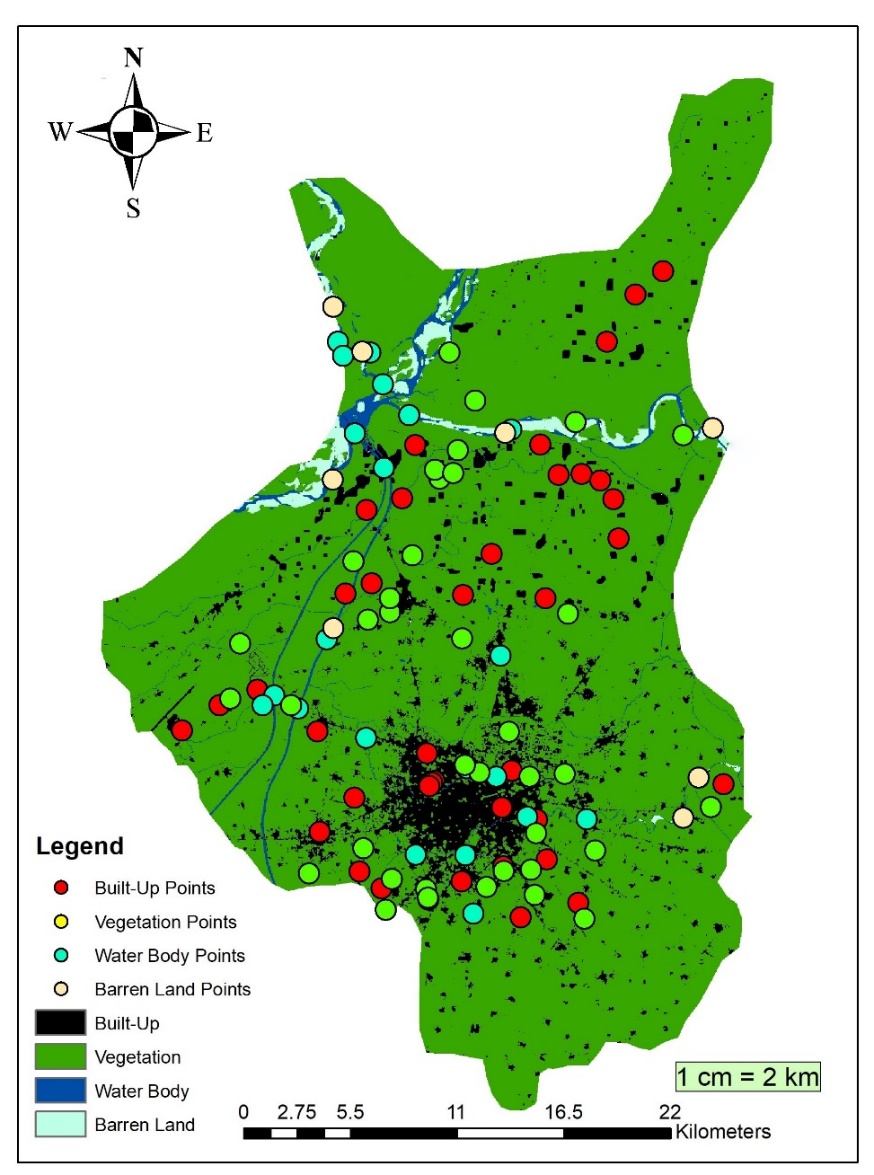


**Fig. SI 2.** Ground truth data for accuracy assessment of predicted map 2030 (Software: ArcMap v. 10.8 & IDRISI SELVA v. 17.0)
